# Health facility capacity and technical efficiency in the provision of adolescent sexual and reproductive health services in Niger

**DOI:** 10.1186/s12889-025-24442-0

**Published:** 2025-09-30

**Authors:** Nassirou Ibrahim, Roxane Borgès Da Silva, Akua Irene Agyepong, Jacob Novignon, Tim Ensor

**Affiliations:** 1 Ecole de Santé Publique de l’Université de Montréal (ESPUM), Montréal, Canada; 2 Laboratoire d’études et de recherches sur les dynamiques sociales et le développement local (LASDEL), Niamey, Niger; 3 Ghana College of Physicians and Surgeons (GCPS), Accra, Ghana; 4 Department of Economics, Kwame Nkrumah University of Science and Technology, Kumasi, Ghana; 5 University of Leeds, Leeds, UK

**Keywords:** Adolescents, Sexual and reproductive health services, Technical efficiency, Organisational capacity, Niger

## Abstract

**Background:**

Efficiency in the use of financial and other resources for providing adolescent sexual and reproductive health (ASRH) policies and programs is an important factor that can affect provision of and access to services in resource-constrained contexts of developing countries with limited capacity. However, very few studies have been conducted to understand this situation. Our study, therefore, estimated technical efficiency scores for health facilities that offer primary ASRH care services in Niger and the relationship between the capacity of these health facilities and their level of technical efficiency.

**Methods:**

The data used for this study were collected from a survey of 71 primary healthcare facilities providing ASRH in Niger from January 28 to March 15, 2022. A stochastic frontier analysis technique based on the Cobb-Douglas production function specification was used for analysis. A Tobit model estimation was used to examine the relationship between health facility capacity and the level of technical efficiency.

**Results:**

The average technical efficiency in production of primary care ASRH services of the health facilities in the sample was 58% implying high levels of technical inefficiency. Disaggregated analysis revealed that the average score was greater in health facilities where the primary caretaker or head of the institution was female (60%) rather than male (53%). Primary healthcare facility capacity was assessed in four dimensions of operational capacity, managerial capacity, adaptive capacity and leadership capacity. There was a positive association between health facility capacity and the technical efficiency score. However, the levels of association differed from one dimension of health facility capacity to another.

**Conclusions:**

There is much room for improving the efficient use of financial and other resources in primary healthcare facilities that provide ASRH in Niger. Potential interventions include giving women more responsibility for these facilities and strengthening the ASRH production capacity of these facilities.

## Background

In Niger, the contribution of adolescent girls to total fertility is approximately 16% [1]. With an average age at first sexual intercourse of 15 for girls and 20 for boys, adolescents are sexually active in the country, and more than one third (1/3) of adolescent girls have had at least one child before their seventeenth birthday. Although adolescents have good knowledge of contraceptive methods (76% for boys compared with 82% for girls), the use of these methods is very low [1, 2]. Only 1.3% of adolescents used at least one contraceptive method, compared with a national average of 8% [2, 3].

Since the International Conference on Population and Development (Cairo, 1994), Niger has been committed to implementing interventions to improve the supply of adolescent sexual and reproductive health (ASRH) services. Youth and adolescent centres were established in 2000 to promote and care for adolescents, particularly sexual and reproductive health (SRH). In 2005, counselling centres were opened in all regions of Niger under the direction of the Ministry of Youth and with support from the United Nations Population Fund United (UNFPA). These centres offer recreational activities and advice to adolescents, particularly on issues related to SRH [4, 5]. To further strengthen this service, the government introduced an Adolescent Friendly Program between 2006 and 2012, under which some forty health facilities were certified as adolescent-friendly health facilities. Providers in these facilities have been trained to address the specific SRH needs of adolescents [5]. After this program ended in 2012, the government has continued to provide regular training for primary care health facility providers on the provision of specific services for adolescents because of the many observed benefits the program brought in terms of managing ASRH-related problems. These benefits include advice on the use of contraceptive methods and confidentiality in the provision of services to adolescents [5–7].

ASRH interventions received financial support from Niger’s technical and financial partners. These include United Nations agencies such as the World Bank, UNICEF and UNFPA; foundations such as the Bill & Melinda Gates Foundation; research centres such as Canada’s International Development Research Centre (IDRC) and the United Kingdom’s Medical Research Council (MRC); and foreign governments through institutions and agencies such as the Spanish Cooperation, the French Development Agency (AFD), Pathfinder and the International Planned Parenthood Federation (IPPF) [8]. Due to these efforts, the provision of ASRH services has improved [4, 6]. However, despite the financial resources received to make SRH services available, the levels of indicators for the supply of services remain less than desired among adolescents [3–6]. In 2017, for example, only 29% of facilities in Niger offered family planning services to unmarried adolescents, compared with 54% in Ghana and 70% in Burkina Faso [6].

Recent studies show that organisations that implement ASRH interventions, whether public or private, face numerous obstacles that negatively affect the production and use of these services [4, 5, 9, 10]. As a result, access to sexual and reproductive health services is still a problem for adolescents [5, 10]. The efficient use of resources is one of the main obstacles to implementing these interventions [5, 9, 10]. Several studies have argued that the provision of healthcare services is a process involving the rigorous and efficient use of available resources, requiring a combination of technical, managerial, adaptive and leadership skills [11–16]. However, very few studies have examined the efficiency in the use of resources for the provision of ASRH services in developing countries. Similarly, few studies have analysed the correlation between the capacity of health facilities and the efficient use of available resources to provide adolescents with SRH services. In Niger, no study on these issues is yet available. With the aim of providing evidence to improve the efficiency of primary healthcare services in resource-constrained developing countries, this study assessed the technical efficiency of health facilities in delivering sexual and reproductive health services to adolescents in Niger.

Several works [17–23] distinguish three types of efficiency: technical, allocative and economic. Technical efficiency (TE) is the ability of a healthcare organisation to provide the maximum number of services from a given set of inputs. Allocative efficiency (AE) expresses the capacity of an organisation to use inputs in optimal proportions, considering their respective costs. Economic efficiency (EE), being the product of the first two, designates the organisation’s capacity to provide the maximum number of services from an optimal use of resources, considering the costs of inputs and the prices of outputs. While determining TE only requires information on inputs and outputs, AE requires information on input costs and output prices, which are difficult to assess [24, 25], even more difficult in low and middle income countries, where data are often unavailable and of limited reliability. In this study, our focus was on technical efficiency.

## Methods

Two approaches are most often used to estimate the technical efficiency scores of decisions making units: a nonparametric approach and a parametric approach [19, 25]. An example of the nonparametric approach is the data envelopment analysis (DEA*).* The DEA uses mathematical programming to construct a nonparametric frontier. In this approach, no production function is needed, only input and output data. Its main drawback is that it attributes all deviations from the frontier to technical inefficiency, without regard for possible influence of measurement error and other random events. The parametric approach uses stochastic frontier functions to estimate input parameters and calculates technical efficiency scores. Its advantage lies in the fact that the functions used to account for measurement errors and other random events [19]. This study used the stochastic frontier analysis (SFA) to compute technical efficiency.

### Data

For this study a total of 71 health facilities were sampled from an initial survey of 250 health facilities. The reason for the sampling is because this study focuses on a particular level of health facilities that provide similar output and use similar resources. Also, facilities that met the inclusion criteria i.e. health facility had offered ASRH services in Niger between 2018 and 2021 but did not have conclusive data were removed in Tobit regression.

The data were collected using a questionnaire developed using two tools: Office of Adolescent Health (OAH) of the Department of health & human services-USA questionnaire [26] and Levesque et al. [27] questionnaire. The OAH questionnaire was designed as part of programmes to prevent teenage pregnancy. The aim of this questionnaire is to measure and evaluate the capacities of organisations that have implemented ASRH interventions. The questionnaire developed by Levesque et al. [27] was based on scientific and grey literature. It was designed to measure the characteristics of healthcare organisations and evaluate the healthcare services provided to the population.

To align certain questions with research objectives and the context, some adjustments were made. The questionnaire was first submitted to a scientific committee at the University of Montreal’s School of Public Health, which conducted a scientific evaluation of the content. After taking their observations into account, the questionnaire was submitted to the *Comité d’éthique de la Recherche en Sciences et en Santé* (CERSES) of the University of Montreal and the *Comité national d’éthique pour la recherche en santé du Niger* (CNERS) for review. After review, both committees gave their ethical approval.

To reinforce its validity, we submitted the questionnaire for evaluation to senior officials from Niger’s Ministry of Public Health, Population and Social Affairs and researchers from the Laboratoire d’études et de recherches sur les dynamiques sociales et le développement local (LASDEL), who have a better understanding of the study context. The latter then made observations and criticisms based on their knowledge and experience. Their observations and criticisms were discussed and amended during an initial working session at the Directorate of Adolescent and Youth Sexual Health of the Ministry of Public Health, Population and Social Affairs and then during a second working session at LASDEL with the researchers.

These working sessions helped to strengthen the internal validity of the questionnaire, as well as the construct validity of some of our key concepts, namely: ‘organisational capacity’, ‘resource mobilisation’, ‘resource utilisation’ and ‘adolescent sexual and reproductive health services/activities’. Once the questionnaire had been validated, an electronic data collection application was used with the CSPro.7.7 software. So, the data was collected using tablets and smartphones and synchronised daily on a server designed for receiving data. This made it possible to regularly monitor the collected data and ensure consistency and quality. Research assistants with at least a bachelor’s degree were recruited and trained to support the data collection.

### Analysis

#### Stochastic frontier model

The Stochastic frontier analysis is attributed to Aigner et al. [28] and Meeusen and Van den Broeck [29]. The approach decomposes the error term in an organization’s production function into two parts, ${\varepsilon _i}={v_i}+( - {u_i})$ in which one of the error terms (${v_i}$) is associated with random shocks and the other ($- {u_i}$) is associated with technical inefficiency of the production process. For cross-sectional data, the stochastic frontier model is expressed as:

1 $${Y_i}=f({X_i};\beta ).exp({\varepsilon _i})$$

Since ${\varepsilon _i}={v_i}+( - {u_i})$, the expression in (1) can be re-written as:

2 $${Y_i}=f({X_i};\beta ).exp({v_i}).exp( - {u_i})$$

Where $\:{Y}_{i}$ is the output level (here, it is the number of sexual and reproductive health consultations made by adolescents at the health facility) for health facility i, $\:f({X}_{i};\beta\:)$ denotes the production function with a vector of inputs $\:{X}_{i}$ (see Table 1), a vector β of parameters to be estimated,${v_i}$ is the random error term and$( - {u_i})$ is a nonnegative random variable associated with specific factors that cause the ith health facility not to reach its maximum potential output.

An important component of the SFA approach is the choice of a production functional form to be estimated. For this study, we chose a Cobb Douglas production function, due to its flexibility and suitability for small sample sizes. In addition, the result test nullity (Likelihood-ratio test) of the coefficients (${\beta _{ij}}$) of all terms of the square and cross products of the Translog form does not allow to reject the null hypothesis (${H_0}:{\beta _{ij}}=0$) at the 5% threshold.

Thus, Eq. (2) was re-specified in the following form.

3 $${Y_i}=f({X_i};\beta )=({\beta _0}\prod\limits_{{i=1}}^{N} {X_{i}^{{{\beta _i}}})} .exp({v_i}).exp( - {u_i})$$

where $\:{X}_{i}$ is the input vector (see Table 1), $\:{\beta\:}_{i}$ the input parameters to be estimated, and N is the number of inputs.

The estimable form of Eq. (3) requires a logarithmic transformation of the variables to obtain the linear function as shown in Eq. (4).

4 $$\ln ({Y_i})=\ln {\beta _0}+\sum\limits_{{i=1}}^{N} {{\beta _i}ln({X_i})} +({v_i} - {u_i})$$

where *ln*^1^ is the natural logarithm. The estimation of the parameters of the Eq. (4) is dependent on the distributional assumptions concerning the two error terms. It is generally assumed that the first error term $$\:{v}_{i}$$ is independently, identically and normally distributed with zero mean and constant variance, $$\sigma_v^2\left[v_i\sim N\left(0,\;\sigma_v^2\right)\right]$$. For the second error term $$\:{u}_{i}$$, different distributions can be assumed with varying specifications. However, this study adopted the model of Battese and Coelli [28, 30], which assumes that $${u_i}$$ follows a normal distribution with mean, i, and variance, $$\sigma _{u}^{2}$$$$[{u_i}\sim N\left( {i,\sigma _{u}^{2}} \right)]$$.

To measure technical efficiency, it is important to separate the composite error term. Jondrow et al. [31] were the first to propose calculating the inefficiency term as a conditional expectation. Other authors have proposed variations on this approach, notably the Battese and Coelli estimator [30]. The Battese and Coelli (BC) approach is relatively simple to implement and understand. The BC model uses the maximum likelihood method to estimate the parameters of the production function, considering both technical inefficiency and random errors. Using numerical optimisation techniques, such as the Newton-Raphson method, it offers a robust approach to estimating the stochastic frontier, particularly in the presence of endogenous explanatory variables [32]. In practice, the BC estimator is available in statistical software such as Stata and R, which simplifies its application in empirical studies [33, 34]. For these reasons, we used the BC estimator in this study to predict the technical efficiency scores of health facilities. The predicted values are between 0 and 1. A value close to 0 means that the health facility is technically less efficient in the use of available resources (inputs) to provide sexual and reproductive health outputs for adolescents. On the other hand, a value close to 1 indicates that the health facility is technically more efficient.

#### Model Tobit

To estimate the association between health facility capacity and the level of technical efficiency, we used the Tobit model which belongs to the family of models with limited dependent variables. The Tobit model belongs to the family of limited dependent variable models. In these models, the dependent variable is continuous but observable only over a certain interval. These models are halfway between qualitative variable models and linear regression models, where the endogenous variable is continuous and observable. The Tobit model was introduced by Tobin in 1958. We chose this model for this study because our dependent variable, technical efficiency score is continuous and takes values in the interval [0, 1] [35–37]. Indeed, this is what econometric literature recommends, as it is a censored variable limited to 1 [38–40]. Estimating the parameters using the ordinary least squares method would lead to a biased estimate, as it assumes a normal and homoscedastic distribution of errors and efficiency scores, which is not the case with Tobit estimation [39]. The model specification is written as the latent regression model with a continuous outcome that is either observed or unobserved. The result for a health facility i is defined as follows:

5 $$ET_{i}^{*}=\left\{ {\begin{array}{*{20}{c}} {E{T_i}} \\ 0 \\ 1 \end{array}} \right.\begin{array}{*{20}{c}} {si} \\ {si} \\ {si} \end{array}\begin{array}{*{20}{c}} {0<E{T_i}<1} \\ {E{T_i} \leqslant 0} \\ {E{T_i} \geqslant 1} \end{array}$$

Where a is the lower limit of censoring and b is the upper limit of censoring.

The Tobit model assumes that the error term is normally distributed; $\varepsilon \sim N{\text{ }}\left( {0,{\text{ }}{\sigma ^2}I} \right)$. Depending on the problem to be solved, the quantity of interest in a Tobit model is the censored outcome, $ET_{i}^{*}$, or the uncensored result, $E{T_i}$. In the scenario of the above measuring instrument, we may wish to predict values that fall below the measurement threshold.

The estimated model is written as follows:

6 $$ET_i=X_i\beta^\prime+\varepsilon_i^\prime$$

Where $E{T_i}$ is the technical efficiency score of health facility ii, ${X_i}$ the vector of independent variables (see Table 1), $X_i$ the vector of parameters associated with the vector of variables${X_i}$ and $\varepsilon _{i}^{\prime }$ error term.

To examine the effect of each dimension of health facility capacity, five models were estimated. The first model (M1) considers all dimensions of facility capacity. The other four (i.e., M2, M3, M4 and M5) consider only one of the four dimensions of health facility capacity, which are managerial capacity, operational capacity, adaptive capacity and leadership capacity, respectively.

### Variables and measurements

#### The production function

##### Output

The number of adolescent sexual and reproductive health consultations recorded by the health facility was used as the output variable in the estimation of the production function. The variable was logarithmic transformed to obtain a linear form while this variable does not capture outcomes beyond the health facility, it presents an opportunity to compare health facilities as it captures the care process under the control of the health facilities. The choice of “number of adolescent sexual and reproductive health consultations” as output was initially based on the literature. Indeed, several studies had also used the number of consultations or visits recorded in health facilities as an output or dependent variable to determine the technical efficiency scores of health facilities, particularly in developing countries where data quality is not always guaranteed [41–43]. Otherwise, indicators relating to patient outcomes or satisfaction could be used as outputs, but information on these indicators was not available at the health facilities visited.

##### Inputs

*A* total of five inputs were used in the estimation of the production function. These include the number of healthcare providers trained in ASRH and available in 2021, the amount of equipment available for ASRH services, the number of condoms available in 2021, the number of pills used available in stock in 2021, and the number of injectable available in 2021. As with the output, all input variables were logarithmically transformed to obtain their linear form. Similarly, the choice of input indicators was based first on literature and then on the availability of information enabling many structures with complete information on inputs and outputs to be identified to conduct relevant technical efficiency analysis. To complete the model, other variables with a potential influence on the technical efficiency of health facilities were used in the model. These variables are sociodemographic characteristics of health facilities and contextual factors (see Table 1).

### Tobit model

#### Dependent variable

The technical efficiency score predicted from the production function were used as the dependent variable for the Tobit model.

#### Independent variables

The main independent variables were the capacities of the primary healthcare facilities. They were operationalised in four dimensions: (i) operational capacity, (ii) managerial capacity, (iii) adaptive capacity and (iv) leadership capacity. Multiple correspondence analysis was used to generate the level of capacity for each of the four dimensions and a composite variable for the level of health facility capacity.

Operational capacity was measured with questions relating to the availability of staff, skills, space and funding needed to offer ASRH services within the health facility. A set of ten questions was posed to each health facility to identify this dimension.

Managerial capacity was measured with questions relating to the existence and implementation of a plan, strategy or procedure enabling the health facility to make efficient use of the resources available to offer sexual and reproductive health services to adolescents.

Adaptive capacity was measured with questions relating to the existence of a data collection system to ensure that the services offered met the needs of the population and that an improvement in the well-being of beneficiaries was observed. Questions on the capacity of health structures to adjust in the event of shocks or unforeseen events were also used to measure this dimension.

Leadership capacity was measured with questions relating to the involvement of health facility managers in the process of offering services. For example, this involves knowing whether the health facility’s management team is actively involved in guiding and inspiring the players to achieve the objectives set.

The composite variable, the level of health facility capacity, was created using all the questions across the four dimensions using principal component analysis. All the questions used to construct the different variables operationalising the four dimensions of health facility capacity are listed in the appendix.

#### Control variables

Several other variables were considered in the Tobit model as control variables (see Table 1). These variables made it possible to control for the influence of organisational capacity variables on the technical efficiency score or to consider the confounding effect of these variables. They were selected based on a review of the literature on the use of resources and the availability of information in the database used.

**Table 1:** Description of analysis variables

Variables/Methods	Description	Stochastic frontier model		TOBIT regression model	
		Considered	*N*	Considered	*N*
External/independent					
ln (Number of sexual and reproductive health consultations by adolescents in 2021)	Continue	Yes	71	No	
Technical efficiency score	Continue	No		Yes	59
Inputs/independent/controls					
ln (Number of providers trained in ASRH and available in 2021)	Continue	Yes	71	No	
ln (Amount of equipment for ASRH services in 2021)	Continue	Yes	71	No	
ln (Number of condoms available in 2021)	Continue	Yes	71	No	
ln (Number of pills available in 2021)	Continue	Yes	71	No	
ln (Number of injectable available in 2021)	Continue	Yes	71	No	
Place of residence	1. Urban 0. Rural	No	71	Yes	59
Geographical area	1. Agadez-Tahoua	No	71	Yes	59
	2. Dosso-Niamey-Tillabéri				
	3. Diffa-Maradi-Zinder				
Gender of health facility head	1.Male	No		Yes	59
	0. Women				
A room/space reserved for teenagers	1. Yes 0. No	No		Yes	59
Place of ASRH within the health facility	1. SSRA is a priority 0. SSRA is not a priority	No		Yes	59
Health structure’s vision of access to ASRH services	1. Absolute right 0. Not at all an absolute right	No		Yes	59
Type of governance	0. Vertical 1. Horizontal	No		Yes	59
Approach to service delivery at the health facility	1. Application of standard 0. Participatory approach	No		Yes	59
Involvement of the community served in the activities of the health facility	1. Yes 0. No	No		Yes	59
Knowledge of the adolescent population in the catchment area	1. Yes 0. No	No		Yes	59
Knowledge of the population in the service area	1. Yes 0. No	No		Yes	59
Radius of coverage	1. Less than 1 km 2. 1–5 km 3. 6 km or more 4. Don’t know	No		Yes	59
Level of mobilisation of financial resources	1. Less than 50 0. 50% or more	No		Yes	59
Number of ASRH jobs available	Continue	No		Yes	59
Number of days ASRH services provided	Continue	No		Yes	59
Level of the health facility’s capacity	Continue	No		Yes	59
Level of the health facility’s managerial capacity	Continue	No		Yes	59
Level of the health facility’s operational capacity	Continue	No		Yes	59
Level of the health facility’s adaptive capacity	Continue	No		Yes	59
Level of the health facility’s leadership capacity	Continue	No		Yes	59

Source: Authors based on survey data from 2022. *N* number of observations

### Testing the validity of the models

A likelihood ratio (LR) test was performed to determine the appropriate functional form used for the production function. Then, a chi-square test was performed to determine whether the presence of inefficiencies in the data was justified.

## Results

### Technical efficiency scores for health facilities

As shown in Table 2, the technical efficiency score ranged between 0.6% and 86.7% with an average score of 58%. However, variations are observed depending on certain characteristics of health facilities. For example, it was greater in health facilities where a woman was the primary facility manager (60%) than in those where the primary facility manager was a man (57%). The average technical efficiency score was also greater for facilities located in urban areas (59.5%) than their counterparts in rural areas (56.7%).

**Table 2:** Technical efficiency scores by health facility characteristics

Characteristics	Obs.	Average	Standard deviation	[95% conf. interval]
Gender of health facility head				
Female	20	0.599	0.267	[0.474 0.724]
Male	39	0.570	0.252	[0.488 0.651]
Place of residence				
Rural	33	0.567	0.244	[0.481 0.654]
Urban	26	0.595	0.273	[0.485 0.705]
Community involvement				
Not regularly	32	0.591	0.271	[0.493 0.688]
Regularly	27	0.566	0.239	[0.472 0.661]
Type of governance				
Vertical	17	0.681	0.225	[0.566 0.797]
Horizontal	42	0.538	0.257	[0.458 0.619]
A room or area reserved for teenagers				
Yes	23	0.705	0.222	[0.493 0.719]
No	36	0.537	0.254	[0.477 0.649]
All facilities	59	0.580	0.255	[0.513 0.646]

Source: Authors based on survey data from 2022

Similarly, as shown on the map below, there were variations between regions, with the average technical efficiency score slightly higher in the regions of Zinder (76%) and Diffa (73%). The highest score was recorded in the Tillabéri region with a value of 20%. Source: Authors



### Determinants of technical efficiency in health facilities

Five models were estimated to analyse the determinants of the technical efficiency of health facilities. The results indicate that these models were all significant at the 5% level and that the Pseudo-R values^2^ represented 20.92%, 13.13%, 12.30%, 19.44% and 13.96% for models M1, M2, M3, M4 and M5, respectively. This indicated that there was at least one variable in each estimated model that explained the technical efficiency score and that the models were predictive. The tests of the presence or absence of inefficiency by the unconstrained models indicated that the hypothesis of the presence of technical inefficiencies was not violated. Table 3 presents the results of estimating the parameters of the Tobit model to explain the technical efficiency score of the health facilities that offered sexual and reproductive health services to adolescents. The parameters can be divided as those related to capacity and those related to other factors.

### Capacity of facilities and technical efficiency score

As shown in Table 3 for Model M1, the coefficient associated with the level of capacity of health facilities was positive and significant at the 1% level. This implies that when health facilities increased their level of capacity by one unit, their technical efficiency score increased by 1.5%, ceteris paribus. However, when the analysis was disaggregated into the four dimensions of health facility capacity (Models M2, M3, M4 and M5 in Table 3), the results indicate that these dimensions did not have the same effect on the technical efficiency score. For example, when health facilities improved their managerial capacity by one 1%, the technical efficiency score increased by 2.2%. This increase was 6.9% and 5.5% when facilities improved their adaptive capacity and leadership capacity, respectively. However, when health facilities improved their operational capacity, there was no effect on the technical efficiency score.

These results showed that the capacity of health facilities determines their ability to efficiently use available resources to offer the maximum number of services to meet the sexual and reproductive health needs of adolescents; with managerial capacity being the most important.

### Other factors and technical efficiency score

Other factors included the area of health facility location (urban or rural), the gender of the primary facility manager, the existence of a space or room reserved for adolescents and the level of mobilisation of financial resources to provide adolescents with SRH services. Particularly in Model M1, the results indicated that health facilities run by women were technically more efficient than those run by men. Similarly, compared with facilities in rural areas, those in urban areas were technically more efficient at using available resources to provide SRH services to adolescents.

Additionally, the existence of a space or room reserved for adolescents’ SRH needs, the adoption of horizontal (or bottom-up) governance and the fact that the health facility considers access to services to be an absolute right were factors that favoured the efficient use of resources to offer adolescents the sexual and reproductive health services they needed.

Furthermore, organisations that mobilised at least 50% of the planned financial resources to implement an ASRH intervention during the 2017–2021 period were technically more efficient than those that mobilised less than 50% of the planned financial resources. Furthermore, as presented in Table 3, the factors identified did not have the same effects on the technical efficiency score, especially when considering the specific models M2, M3, M4 and M5.

**Table 3:** Results of the Tobit model estimates of the technical efficiency scores of health facilities offering ASRH services in 2021

Variables/Methods	M1: Global model			M2: Cap. Management			M3: Cap. Operational			M4: Cap. Adaptive			M5: Cap. In leadership		
	Coef.	[95% CI]		Coef.	[95% CI]		Coef.	[95% CI]		Coef.	[95% CI]		Coef.	[95% CI]	
Geographical area															
Agadez-Tahoua	Ref.			Ref.			Ref.			Ref.			Ref		
Dosso-Niamey-Tillabéri	0.108	[−0.026	0.242]	0.121*	[−0.024	0.265]	0.126*	[−0.024	0.276]	0.065	[−0.046	0.175]	0.144**	[0.005	0.283]
Diffa-Maradi-Zinder	0.271***	[0.102	0.439]	0.308***	[0.128	0.489]	0.287***	[0.095	0.479]	0.204***	[0.064	0.344]	0.331***	[0.157	0.505]
Place of residence															
Rural	Ref.			Ref.			Ref.			Ref.			Ref.		
Urban	0.117**	[0.005	0.229]	0.084	[−0.036	0.205]	0.054	[−0.068	0.177]	0.118**	[0.030	0.207]	0.091	[−0.023	0.206]
Gender of health facility head															
Woman	Ref.			Ref.			Ref.			Ref.			Ref.		
Men	0.033	[−0.057	0.123]	0.045	[−0.053	0.143]	0.018	[−0.053	0.143]	0.003	[−0.071	0.077]	0.050	[−0.044	0.145]
Type of governance															
Vertical	Ref.			Ref.			Ref.			Ref.			Ref.		
Horizontal	−0.200***	[−0.332	−0.069]	−0.162**	[−0.301	−0.022]	−0.157**	[−0.304	−0.010]	−0.251**	[−0.360	−0.142]	−0.166**	[−0.300	−0.032]
Place of ASRH within the health facility															
ASRH is not a priority	Ref.			Ref.			Ref.			Ref.			Ref.		
ASRH is a priority	0.091	[−0.049	0.232]	[0.060	[−0.093	0.212]	0.074	[−0.083	0.232]	0.149	[0.033	0.265]	0.138*	[−0.012	0.289]
Health structure’s view of access to ASRH services															
Not at all an absolute right	Ref.			Ref.			Ref.			Ref.			Ref.		
Absolute right	0.168***	[0.052	0.284]	0.177***	[0.051	0.303]	0.157**	[0.027	0.287]	0.130***	[0.035	0.225]	0.169***	[0.048	0.290]
Approach to service delivery at the health facility															
A participatory approach	Ref.			Ref.			Ref.			Ref.			Ref.		
Application of standards	0.261***	[0.134	0.388]	0.266***	[0.128	0.403]	0.212***	[0.062	0.361]	0.288***	[0.184	0.392]	0.290***	[0.156	0.425]
Involvement of the community served in the activities of the health facility															
No	Ref.			Ref.			Ref.			Ref.			Ref.		
Yes	0.099*	[−0.010	0.209]	0.075	[−0.043	0.193]	0.100	[−0.025	0.225]	0.107**	[0.018	0.196]	0.100*	[−0.014	0.214]
Knowledge of the adolescent population in the catchment area															
Yes	Ref.			Ref.			Ref.			Ref.			Ref.		
No	−0.176***	[−0.269	−0.083]	−0.132***	[−0.228	−0.036]	−0.119**	[−0.222	−0.016]	−0.206***	[−0.281	−0.131]	−0.142***	[−0.234	−0.050]
Knowledge of the population in the service area															
No	Ref.			Ref.			Ref.			Ref.			Ref.		
Yes	−0.095	[−0.251	0.061]	−0.093	[−0.262	0.076]	−0.104	[−0.283	0.074]	−0.058	[−0.185	0.070]	−0.065	[−0.228	0.097]
Radius of coverage															
Less than 1 km	Ref.			Ref.			Ref.			Ref.			Ref.		
1-5Km	0.182**	[0.014	0.349]	0.209**	[0.029	0.390]	0.204**	[0.016	0.391]	0.126*	[−0.012	0.264]	0.186**	[0.012	0.360]
6 km and more	0.343***	[0.169	0.517]	0.372***	[0.182	0.561]	0.337***	[0.142	0.532]	0.239***	[0.094	0.384]	0.368***	[0.186	0.550]
Don’t know	0.043	[−0.094	0.180]	0.053	[−0.096	0.203]	0.032	[−0.122	0.185]	0.009	[−0.102	0.121]	0.025	[−0.117	0.168]
A room/space reserved for teenagers															
No	Ref.			Ref.			Ref.			Ref.			Ref.		
Yes	0.268***	[0.167	0.369]	0.291***	[0.183	0.400]	0.293***	[0.178	0.408]	0.232***	[0.149	0.316]	0.317***	[0.214	0.419]
Level of mobilisation of financial resources															
50% or more	Ref.			Ref.			Ref.			Ref.			Ref.		
Less than 50	−0.116**	[−0.218	−0.014]	−0.111*	[−0.222	−0.001]	−0.121**	[−0.240	−0.001]	−0.039	[−0.124	0.045]	−0.119**	[−0.226	−0.012]
Number of employees trained in ASRH available	−0.014	[−0.035	0.008]	−0.021**	[−0.044	0.002]	−0.021*	[−0.045	0.003]	0.003	[−0.016	0.021]	−0.009	[−0.032	0.014]
Number of days ASRH services provided	0.0001	[−0.030	0.030]	0.005	[−0.028	0.037]	0.003	[−0.031	0.037]	−0.006	[−0.031	0.018]	−0.010	[−0.042	0.022]
Level of the health facility’s capacity	0.015***	[0.008	0.023]												
Level of the health facility’s managerial capacity				0.022**	[0.005	0.040]									
Level of the health facility’s operational capacity							0.023	[−0.008	0.053]						
Level of the health facility’s adaptive capacity										0.069***	[0.050	0.087]			
Level of the health facility’s leadership capacity													0.055***	[0.023	0.088]
Constant				−0.151	[−0.434	0.281]	0.098	[−0.260	0.456]	−0.151	[−0.415	0.114]	−0.231	[−0.602	0.140]
Variance in technical efficiency (e. TE)	0.010***	[0.007	0.015]	0.020***	[0.014	0.029]	0.022***	[0.015	0.031]	0.011***	[0.008	0.017]	0.019***	[0.013	0.027]
Number of obs.	59			59			59			59			59		
LR chi2(22)	109.31***			68.61***			64.25***			101.58***			72.91***		
R2 username	20.92			13.13***			12.30***			19.44***			13.96***		

Source: Authors based on survey data from 2022. ***, **, and * indicate significance at 1% (*p*<1%), 5% (*p*<5%) and 10% (*p*<10%), respectively. M1: First model 1; M2: Second model; M3: Third model; M4: Fourth model; M5: Fifth model

## Discussion

Efficient resource utilization in implementing Adolescent Sexual and Reproductive Health (ASRH) interventions is one of the key factors affecting the effectiveness of policies and programs aimed at improving adolescent health and well-being in developing countries [2, 4, 5, 8, 9]. While some organisations managed to make efficient use of the financial resources received to implement ASRH interventions, others, such as health facilities, struggled to do so. Given limited resources, enhancing efficiency remains crucial—particularly in the resource-constrained environments found in low- and middle-income countries. According to the results, the average technical efficiency score for health facilities offering ASRH services was 58%. This means that around 42% of the number of adolescent sexual and reproductive health consultations were not carried out because of the technical inefficiency of the health facilities. Given that the efficiency score varied between 0.6% and 86.7%, we could conclude that the health facilities had the possibility of improving the recorded number of ASRH consultations, although it would not be possible to recover all the remaining 42% of efficiency score. These results corroborate those obtained by Novignon and Nonvignon in their work in Ghana [44] Kasa, Bowser and Nandakumar in Haiti [42] and Binyaruka and Anselmi in Tanzania [45].

Disparities emerged when the analysis was disaggregated according to certain characteristics of the health facilities as the location area. Facilities in urban areas had a higher efficiency score (59.5%) than those in rural areas (56.6%). Like the area of residence, the average technical efficiency score differed from one zone to another. It was 76% and 73% respectively in the Zinder and Diffa regions, but slightly lower in Tillabéri (20%) or Tahoua (52%). These differences may be related to factors such as increased supervision and resource oversight by health authorities in urban areas [46–49]. Structural factors such as differences in the allocation of human and material resources could also explain these disparities [46–50].

These disparities were also observed when the analysis was specified to other health facility characteristics such as the existence of a space or room reserved for adolescents, the type of governance of the health facilities, the health facilities’ vision of adolescent access to SRH services or the approach to SRH service provision adopted by the health facilities. However, these results could prove to be misleading, which is why we carried out an explanatory analysis using estimates from the Tobit model, focusing on the relationship between organisational capacity and the efficiency score, which was one of the objectives of this article.

The results showed a positive relationship between the capacity of health facilities and their technical efficiency score. More specifically, when health facilities simultaneously improved all four dimensions of their capacity level by one unit, their technical efficiency score increased by 1.5%. This increase differed, however, according to the capacity dimension of the facilities in question. It was 2.2% if the health facility improved only its managerial capacity. This proportion was slightly higher when the health facilities improved only its leadership capacity (5.5%) or its adaptive capacity (6.9%). On the other hand, improving operational capacity in isolation did not automatically lead to an increase in the technical efficiency score, all other things being equal. This result confirmed the hypothesis that the use of resources requires compliance with accounting standards and procedures, the applicability of which requires managerial skills, regular monitoring, effective leadership and an adaptive dynamic [51–53]. It was also in line with the approaches developed by Dimoff [54] and Hobfoll [55] or the thinking of Connolly & York [56], Wagner-Rundell & Stratford [57], for whom the managerial, adaptive and leadership capacities of organisations strongly influence the use of resources within them.

In addition, the estimates revealed other factors which explained the variations in the efficiency score of health facilities. These factors included the gender of the person in charge, the type of governance, the health facility’s vision of access to adolescent sexual and reproductive health services, the health facility’s approach to providing SRH services to adolescents, the health facility’s knowledge of the adolescent population in the coverage area, the existence of a space or room reserved for ASRH services, the level of mobilisation of financial resources to implement ASRH interventions and adaptive capacity. However, these factors had varying influences on the technical efficiency score.

Indeed, econometric analyses indicate that health facilities under female management demonstrated greater technical efficiency compared to those led by males. These findings are consistent with our descriptive results and align with the evidence reported by Novignon et al. [7] in Ghana. Although the reason for higher efficiency scores in female-headed health facilities is unclear in the literature because there are limited studies examining the relationship between manager gender and health facility efficiency in developing countries, women’s dynamism and rigour in using available resources to offer the maximum number of healthcare services could be an explanation. Similarly, compared with men, women’s sensitivity to adolescent sexual and reproductive health issues could lead women to make more efficient use of available resources and offer adolescents as many SRH services as possible.

It also appeared that facilities that had a space or room specifically for adolescents were technically more efficient than those that did not. This result could be explained by the fact that facilities with adolescent areas were more aware of sexual and reproductive health issues and could adopt behaviours that would encourage the efficient use of resources to offer the maximum number of services to adolescents [4–6].

The results also indicated that health facilities with knowledge of the adolescent population in the coverage area were more likely to have a higher technical efficiency score than those with no knowledge of this population. According to authors such as Pineault [51], offering services to a population first requires an assessment of their needs. This undeniably means looking at the population’s socio-demographic characteristics, because its needs are a function of its condition and its composition according to characteristics such as age, sex or marital status. Consequently, a health facility that knew the characteristics of the population it served would be more careful in managing its resources, by putting in place a set of tools to ensure better management of available resources and to be able to offer the maximum number of services that the population needs.

The findings further demonstrated that health facilities which had mobilised at least 50% of their planned funds exhibited greater technical efficiency score compared to those achieving less than 50% of the targeted resource mobilisation for sexual and reproductive health services. Resource mobilisation was becoming an increasingly complex area, especially in developing countries such as Niger, and was attracting more players in search of funding. Consequently, this creates a competition between actors in terms of mobilising financial resources intended for ASRH interventions [58–60]. Actors wishing to achieve a high level of financial resource mobilisation were thus forced to make efficient use of resources in order to increase their credibility with donors and gain easier access to funding [60].

### Recommendation 1

Given that approximately 42% of adolescent sexual and reproductive health (SRH) consultations were not conducted due to the technical inefficiency of health facilities—and that Tobit model estimates indicate a positive association between technical efficiency and the capacity to deliver SRH services to adolescents—it is essential to reinforce the capacity of these facilities. This may involve implementing targeted capacity-building sessions for health facility managers and heads, focusing on the efficient utilisation of available resources and appropriate management techniques. Such training can enhance resource management and operational effectiveness. Funding and organisation of these sessions may be undertaken by the Government of Niger or its technical and financial partners, such as the World Bank, UNFPA, Pathfinder International, USAID, and WHO.

### Recommendation 2

Findings indicate that health facilities with dedicated spaces for adolescents experience increased adolescent attendance and more efficient utilisation of financial resources compared to those without such spaces. It is therefore recommended that all health facilities establish areas specifically reserved for adolescents to support greater uptake of sexual and reproductive health services. Government or development partner support is essential to enable health facilities to implement these measures effectively.

### Strengths and limitations

This study investigated the allocation of resources for the provision of Adolescent Sexual and Reproductive Health (ASRH) services, an aspect infrequently explored in developing countries. The findings contribute valuable insights into resource utilisation in such contexts and offer evidence that may be leveraged by stakeholders to enhance support for health facilities delivering ASRH services.

Nevertheless, several limitations should be acknowledged. The survey-based collection method has potential biases, as self-reported data may be influenced by desirability or memory errors. The absence of certain control or confounding factors that could influence the results is also a limitation. Furthermore, the technical efficiency analysis used a Cobb-Douglas function and a process variable as output, which may limit the scope of the conclusions. It would be relevant to conduct another study using a Translog function and a patient outcome or satisfaction variable as output. Qualitative research can provide a more comprehensive understanding of specific relationships, such as those between gender and the technical efficiency of health facilities.

## Conclusions

Resource use in ASRH interventions remains a key challenge. This study investigated how health facility capacity relates to technical efficiency in delivering adolescent SRH services. Findings indicate a positive relationship: facilities that enhance managerial, operational, adaptive, and leadership capacities see higher technical efficiency scores. The strongest correlations were observed with improvements in adaptive, managerial, and leadership areas, while operational capacity showed no significant effect. Thus, boosting these specific capacities is important for effective resource use and improved access to ASRH services.

Other factors also influenced the technical efficiency scores of health facilities, including health facility head gender, residence area, governance type, health facility vision and approach toward adolescent SRH services, knowledge of local adolescents, and the presence of a dedicated ASRH space. The strength of each factor’s correlation with efficiency varied, especially across different dimensions of health facility capacity. The findings offer evidence to support interventions in adolescent sexual and reproductive health. However, further research is needed, such as quantitative or qualitative studies for deeper insight or studies in other developing countries to broaden the results.

## Data Availability

The datasets used during the current study are available from the corresponding author on a reasonable request.

## Footnotes

1 Neperian logarithm or natural **logarithm**.

## Abbreviations

Adowa: Adolescent Health West Africa
AE: Allocative Efficiency
AFD: French Development Agency
ANBEF: Association nigérienne pour le bien-être familial
ASRH: Adolescent and Sexual and Reproductive Health
BC: Battese and Coelli
CERSES: Comité d’éthique de la Recherche en Sciences et en Santé
CNERS: Comité National d’éthique pour la Recherche en Santé du Niger
DEA: Data Envelopment Analysis
EE: Economic efficiency
FDA: French Development Agency
FRM: Fractional Response Model
GLM: generalized linear models
HIV: Human Immunodeficiency Virus
HIV: Human Immunodeficiency Virus
ICPD: International Conference on Population and Development
IDRC: International Development Research Centre
IPPF: International Planned Parenthood Federation
LR: likelihood ratio
MRC: Medical Research Council
NCFH: National Centre for Family Health
OLS: Ordinary Least Squares
QMLE: Quasi-Maximum Likelihood Estimation
RH: Reproductive Health
RNG: Random Number Generator
SFA: Stochastic Frontier Analysis
SRH: Sexual and Reproductive Health
STI: Sexually Transmitted Infection
TE: Technical Efficiency
UK: United Kingdom
UNESCO: United Nations Educational, Scientific and Cultural Organisation
UNFPA: United Nations Fund for Population
UNICEF: United Nations International Children’s Emergency Fund
USAID: United States Agency for International Development
WAHO: West African Health Organisation
WHO: World Health Organisation
